# Ribosome Evolution and Structural Capacitance

**DOI:** 10.3389/fmolb.2019.00123

**Published:** 2019-11-14

**Authors:** Ashley M. Buckle, Malcolm Buckle

**Affiliations:** ^1^Department of Biochemistry and Molecular Biology, Biomedicine Discovery Institute, Monash University, Clayton, VIC, Australia; ^2^LBPA, ENS Paris-Saclay, CNRS, Université Paris-Saclay, Cachan, France

**Keywords:** structural capacitance, ribosome evolution, protein disordered region, disorder-order transition, codon-anticodon, genetic code

## Abstract

In addition to the canonical loss-of-function mutations, mutations in proteins may additionally result in gain-of-function through the binary activation of cryptic “structural capacitance elements.” Our previous bioinformatic analysis allowed us to propose a new mechanism of protein evolution – structural capacitance – that arises via the generation of new elements of microstructure upon mutations that cause a disorder-to-order (D→O) transition in previously disordered regions of proteins. Here we propose that the D→O transition is a necessary follow-on from expected early codon-anticodon and tRNA acceptor stem-amino acid usage, via the accumulation of structural capacitance elements – reservoirs of disorder in proteins. We develop this argument further to posit that structural capacitance is an inherent consequence of the evolution of the genetic code.

## Introduction

Like all cellular life, proteins evolved by Darwinian natural selection. Once a primitive enzyme acquired even very weak catalytic activity, genetic mutations followed by selection, did the rest. But here is the catch – how would classic Darwinian evolution proceed in the absence of pre-existing seed structures and functions? As pointed out by Dan Tawfik, nothing evolves unless it already exists (Tawfik, 2013). Darwinian selection needs something, some function, to select for (or against). When life started more than three billion years ago, what was the spark that created this “something” from randomness, and ignited the evolution of a protein fold?

Becker et al. (2019) have recently demonstrated that pyrimidine and purine bases can be synthesized from small molecules present in the prebiotic environment. Thus, it is extremely likely that RNA was relatively abundant in the prebiotic environment lending great plausibility to the idea of an RNA world that then evolved into an RNA-peptide and RNA-protein world that constituted the precursors to life.

We recently postulated a hypothesis that identifies a mechanism whereby microstructure is generated *de novo* in hitherto disordered regions of protein (Li et al., 2018) This process, termed structural capacitance, provides a mechanism to accelerate Darwinian natural selection by taking advantage of a disorder-to-order (D→O) causing mutation that will generate a function conferring neostructure in a hitherto poorly structured region of a protein. This idea was supported by a thorough database analysis of over 68000 human disease-associated mutations which serve as reservoirs, or “structural capacitance elements” in which a single gene mutation may create nucleating seeds that can act as a “feedstock” for evolution to proceed. Structural capacitance is compatible with emerging evidence for significant structural changes induced by mutation (Arodz and Plonka, 2012; He et al., 2012; Toth-Petroczy and Tawfik, 2014), the “dormant foldon” hypothesis (Uversky, 2013), concepts of early protein evolution driven by peptide-world “foldamers” and Dayhoff's hypothesis (Romero Romero et al., 2016), as well as oligomerization-duplication-fusion events of short peptide (Soding and Lupas, 2003; Chessari et al., 2006).

Here we suggest that the D→O transition is a necessary follow-on from expected early codon-anticodon and tRNA acceptor stem-amino acid usage. Irrespective of how existing codon-anticodon and stem-amino acid usage evolved, our essential hypothesis is that it allowed the accumulation of reservoirs of disorder in proteins (structural capacitance elements) that have been maintained in the present ecosystem.

Many factors are evoked to explain codon-anticodon and stem-amino acid configurations as they exist today; these necessarily represent a compromise between mutational bias and protein translation requirements optimized through natural selection. However, for our purposes we need to wind the clock back to the very early stages in the development of the post RNA, early ribosomal world. We will first think about these two central aspects of ribosomal mechanisms; codon:anticodon interactions and acceptor stem:amino acid recognition.

From a purely chemical point of view, the idea that the stability of codon:anticodon interactions was predominant in the development of the genetic code was initially brokered by Eigen and Schuster (Eigen and Schuster, 1978) and given strong support by the application of the thermostability rule (Trifonov, 2000) to coding triplets determined by the stacking energies of adjacent nucleotides (Krueger et al., 2006; Travers, 2006). Furthermore, it was recently postulated that stem:amino acid recognition is correlated with the size of the amino acid and that codon:anticodon recognition is based on polarity with a strong correlation between polar/non-polar and Purine/Pyrimidine frequency at the 2nd anticodon position (Carter and Wolfenden, 2015).

In the prebiotic world, as originally suggested by Jukes (1967) and Crick (1968), the initial code would have been a 2-letter triplet (XYNXYN…) where the XY base step controlled the codon:anticodon interaction, thus XYN specified a particular amino acid with a usage of GC (Ala) GG/CC (Gly, Pro), GT/AC (Val, Thr), GA/TC (Asp or Glu).

The most stable codon-anticodon pairs would have been selected for the restricted set of amino acids likely to have been present, namely Asp, Glu, Thr Ala, Gly, Val and in decreasing order of polarity (Miller and Urey, 1959). Indeed, all of these amino acids are coded by single changes in the GCU triplet (Trifonov, 2000). Trifonov has established a chronological order of amino acid appearance based on codon:anticodon thermostability; Gly/Ala, Val/Asp, Pro, Ser, Glu/Leu, Thr, Arg, Asn, Lys, Glu, Ile, Cys, His, Phe, Met, Tyr, and Trp (Trifonov, 2000). If the most stable codon:anticodon couples were therefore selected by the simple prebiotic amino acids this suggests an inverse correlation between codon:anticodon stability and amino acid complexity. Thus, at the beginning of the transition from the RNA world one would expect most early proteins to have been of low complexity and thus disordered or poorly structured. Eventually, moving down this list, a critical threshold of amino acids would have been reached, at which point moving to more complex protein structures would be limited by the lack of chemically diverse amino acids. Catalysis is required for efficient replication and, in the RNA world this would have arisen from RNA secondary structure. A current hypothesis is that acceptor stem recognition preceded anticodon recognition (Carter and Wolfenden, 2015) and that this selection, based essentially on size discrimination led to a prevalence of beta structure with alternating small and large amino acids. However, this argument does not address the question of how these amino acetylated tRNA molecules then lined up on an mRNA template in the absence of anticodons.

Independent, however of whichever mechanism arrives first – stem recognition or codon recognition – the limited subset of amino acids originally present (Asp, Glu, Ala, Val, Gly, and Thr of which two are polar/charged, two are non-polar, and two are indifferent, respectively) occupying necessarily the most stable codon:anticodon arrangements argues that the majority of the initial primordial proteins were relatively poorly structured. Indeed, recent findings demonstrated that proteins depleted in cysteines and aromatic residues were intrinsically disordered and yet extensively distributed across all of life (Yan et al., 2019). Of particular interest is that many of these, what the authors colorfully refer to as “non-smelly” proteins, are disordered and interact specifically with nucleic acids and thus are crucial for the basic processes of life such as replication, transcription and chromatin organization and the ensuing epigenetic regulation of gene expression. This adds considerable weight to the hypothesis that early peptides/proteins consisted of a simple subset of amino acids that were therefore intrinsically disordered.

Finally, many biologically active proteins are intrinsically unstructured and yet have functions not associated with catalytic activities. A prime example of such a class would be the histone proteins associated with nucleosome structure (Yan et al., 2019). Indeed, disorder may offer advantages for gene regulation, as in the case of the unstructured dynamic complex between histone H1 and its chaperone ProTα (Borgia et al., 2018); high affinity is achieved by ultra-fast association and is not reliant upon molecular recognition driven by structurally-defined binding sites. Intriguingly, there is some evidence to suggest that disordered segments in proteins act as “entropic rectifiers,” tuning the energy landscape of the entire protein, offering an elegant and simple evolutionary adaptation mechanism (Keul et al., 2018). In the light of our hypothesis, one would predict that such proteins may in fact have been present relatively early and it would be extremely interesting to look at those biological functions associated with disordered proteins in an evolutionary context.

The elephant in the room here is that, as pointed out by Belousoff et al. (2010) the initial proto ribosome would require early peptides to interact with the RNA molecules and for that, the positively charged amino acids (His, Lys and Arg) would be highly selected. However, none of these amino acids are expected to be present in the prebiotic broth. Andrew Travers suggested two possible work arounds for this (Travers, 2006), either the early biotic environment somehow contained sufficient Arg so that it “grabbed” a strong codon:anticodon slot (CGN), or that since one of the products in the Miller reaction was found to be norleucine and that CGN was originally taken by norleucine; subsequent cyanide and ammonia derivation of the unbranched norleucine generated Arg that then displaced the norleucine from the CGA codon:anticodon slot.

It follows therefore that the D→O transition is implicit in the genetic code simply because all the stable codon:anticodon interactions were initially taken by simple amino acids less capable of producing ordered structure. Consequently, any mutation would invariably lead to a less stable codon:anticodon interaction allowing other amino acids to come in and fill the void. Since other amino acids with few exceptions are chemically more complex than the limited prebiotic subset, then this would allow for more and more order to appear in protein structure. Nonetheless early proteins would have had tracts of disorder intrinsically present, with little evolutionary pressure to remove them and huge codon:anticodon pressure to remain!

Eventually, the initial set of proteins resulting from this process and making up the phenotype of a primitive organism would therefore already have a set of structure/function motifs in its proteome within tracts of disorder. Subsequent O→D transitions would thus be probably deleterious if they occurred within the structured folds; D→O transitions could still occur, but would require moving from stable to unstable codon:anticodon interactions. Since this interaction, however, is no longer important in terms of codon usage as the ribosome already exists, the effect would then be simply on protein function, and would therefore lead to structural capacitance. So in other words, the original evolutionary pressure to select codon use (i.e., to select stable codon:anticodon interactions) is no longer present and a consequence of the way that this initially evolved means that all D→O mutations which mostly involve stable to unstable codon:anticodon changes are refractive to evolutionary pressure, *except of course if that pressure is now exerted at the level of the ensuing new fold or structure*. The codon change involved in D→O transitions is neutral as far as selection at the level of codon usage is concerned. Once a primitive ribosome has started to attribute codon usage under the sole selective pressure of codon:anticodon stability, there is no going back. It is remarkable but logical that codon:anticodon stability favors simple amino acids. Structural capacitance therefore is an inherent consequence of ribosome evolution.

At this point it is worth discussing the fascinating results of a study by Root-Bernstein and Root-Bernstein (2015). The authors discuss the origin of ribosomes in terms of three hypotheses: firstly, that ribosomes evolved prior to cellular life and was to a large extent genetically self-sufficient, secondly that rRNA is purely structural (which is the classical text book definition and that they call the null hypothesis) and finally that any genetic information present in ribosomes is purely random. Their study strongly argues for the first hypothesis and we would like to discuss the ramifications of this in terms of structural capacitance and its implicit origin in early codon:anticodon usage. They showed that rRNA regions possessed the capacity to code for all 20 tRNAs in an overlapping fashion. They also showed that rRNA coded for proteins associated with ribosomes and furthermore they identified between 30 and 55% of identified rRNA that encoded proteins with similarities to functional sites of identified active regions of proteins that they mimic, such as polymerases, helicases, phosphodiesterases and so forth.

The presence of intrinsic disorder in ribosomal proteins has been known for a long time. A comprehensive bioinformatics analyses of over 3,000 ribosomal proteins from 32 species shows that intrinsic disorder is very common in ribosomal proteins (Peng et al., 2006). The evolutionary advantage conferred by intrinsic disorder is consistent with the function of ribosomal proteins as interaction hubs requiring promiscuous binding to many different binding partners from the translational machinery that includes both proteins and RNA. In addition, the observed “moonlighting” or off-ribosome functionality of ribosomal proteins (Weisberg, 2008) is consistent with the structural malleability of intrinsically disordered proteins (Tompa et al., 2005). An interesting test of our hypothesis in the light of the Root-Bernstein results would be to examine the structures of ribosomal proteins (and the active sites of proteins sharing homology with rRNA encoded proteins) to determine the extent of structural capacitance present. To investigate this further we interrogated D2Odb, an online resource of predicted structural transitions for mutations in human proteins, which we recently created (http://D2Odb.org; Fulton Buckle *et al*, unpublished). We retrieved all records relating to ribosomal proteins by searching using the terms “ribosomal” or “ribosome” or “tRNA” or “mRNA.” We found a total of 801 mutations within 245 different ribosomal proteins; the majority (567, 71%) are predicted to be O→O transitions, 195 predicted as D→D (24%), 16 predicted as O→D (2%) and 23 predicted as D→O (3%). Although our dataset is focussed exclusively on known disease-associated mutations in human proteins (and thus contains only a subset of all possible mutations), it is clear that many ribosomal proteins harbor mutations that likely alter local structure, including those that may induce structure in disordered regions.

Thus, in existing ribosomes we find that codon:anticodon use is common for both tRNA synthesis from rRNA and protein synthesis from rRNA. Consequently, would this not then argue that codon recognition predates stem recognition since tRNA synthesis from rRNA would not necessarily require proteins? If this were the case then as we argue above, the most stable codon:anticodon pairs *that already exist* would be grabbed by the most abundant amino acids available (D, E, A, G, V, T) with little potential for structure.

This is in fact borne out by an extensive energetic appraisal of codon interactions carried out by Grosjean and Westhof (2016) that supports the concept that complementary RNA:RNA duplexes constituted the original proto ribosome in which stable complimentary G:C rich triplets coded for small polypeptides consisting of alternating Ala and Gly amino acid. As Grosjean and Westhof succinctly explain, weaker codon:anticodon pairs would associate with more diverse emerging amino acids and interestingly enough would therefore constitute a pool of more and more deviations from the genetic code and therefore leave place for the insertion of additional amino acids and thus structure!

As mentioned earlier, one of the factors dictating ribosome function is protein translation requirements, in other words how fast can the protein fold as it peels away from the ribosome. Both sequential and non-sequential folding mechanisms dictate that disordered structures will impose less of a kinetic barrier to this than ordered regions. Thus, the presence of disordered regions will act as “fast lanes” during protein synthesis and there will be a certain selection pressure to maintain them. Another exciting possibility, and here there may be metaphoric parallels with nucleosome organization and the control of gene expression, is that the distribution of these disordered regions in proteins may have been selected to ensure a specific overall rate of synthesis, in other words if synthesis of a particularly highly structured protein becomes economically difficult because of the time limitations on folding then maintaining some optimally spaced disordered regions in the protein to allow the ribosome to put its foot down a bit, will alleviate the road block! Again, of course these are not designed; according to our hypothesis, early proteins would have had huge areas of disorder, these would have been reorganized with selection but many will have been retained precisely for the reason outlined above. Indeed, there is no way of knowing in advance when conditions will arise such that structural capacitance will, through a random mutation in the disordered region, provide a useful fold/function; using disordered regions as “reverse governors” during protein synthesis will ensure their retention in the gene pool.

## Concluding Remarks

Whilst the early codon usage may not be the “best” for the whole range of amino acids it will have dictated that structural capacitance is embedded in the system. In fact, had prebiotic amino acids been more polar and complex, then structural capacitance would not be present and it is highly unlikely that evolution would have the time to have produced the huge range of diversity in life that exists today. A prediction of this is, therefore, if or when we find extra-terrestrial life, it will be complex only if it has evolved from simple structures!

## Author Contributions

AB and MB wrote the paper.

## Dedication

This article is dedicated to the memory of Maurice Buckle whose influence on us both was immeasurable. *Ad inordinationem* Dad.

### Conflict of Interest

The authors declare that the research was conducted in the absence of any commercial or financial relationships that could be construed as a potential conflict of interest.
